# Anti-inflammatory and anti-rejection effects of herbal medicine ingredients in organ transplantation: a systematic review and meta-analysis

**DOI:** 10.3389/fimmu.2025.1568988

**Published:** 2025-06-05

**Authors:** Jingru Lin, Zhennan Wu, Huazhen Liu, Feifei Qiu, Zhenhua Dai

**Affiliations:** ^1^ Immunology Program, the Second Affiliated Hospital of Guangzhou University of Chinese Medicine, Guangzhou, China; ^2^ South China Research Center for Acupuncture, Medical College of Acu-Moxi and Rehabilitation, Guangzhou University of Chinese Medicine, Guangzhou, China

**Keywords:** apoptosis, herbal medicine, immune cells, ingredients, inflammatory factors, transplantation

## Abstract

**Background:**

Although postoperative rejection in transplant patients can be managed with immunosuppressants, their use is associated with some complications due to excessive immunosuppression. Recent animal studies in allotransplantation have suggested that certain ingredients of Chinese herbal medicine can extend transplant survival. However, their effects on transplantation have not been systematically reviewed and analyzed. The aim of this study was to evaluate the effects of herbal medicine ingredients on complications and survival of transplanted organs after heart, liver and kidney transplantation, and to explore the possible mechanism of action.

**Materials and methods:**

Databases, including PubMed, EMBASE, Cochrane Library, Web of Science, Wang Fang, China National Knowledge Infrastructure (CNKI), China Science and Technological Journal Database (VIP) and Chinese Biomedical Literature Database (CBM), were searched up to January 1 2025. Animal studies reporting the effects of Chinese herbal medicine ingredients (HMIs) on postoperative complications and organ transplant survival/outcome were included. Methodological quality was assessed using the SYRCLE risk of bias tool. Meta-analysis was performed using R 4.3 software to assess levels of inflammatory factors, oxidative stress markers, apoptosis markers, indicators of liver/kidney function, median graft survival time and immune cell subsets.

**Results and conclusions:**

A total of 18 studies, involving 357 rodents were included. The overall quality of the included reports was moderate. We found that HMIs enhanced organ graft survival by reducing the Banff score, extending the median survival time (MST), and exerting anti-inflammatory, antioxidant and anti-apoptotic effects. HMIs can also inhibit T cell proliferation, dendritic cell (DC) maturation and increase the proportion of CD4^+^ regulatory T (Treg) cells. Furthermore, the improvement in liver and kidney function indicators, such as alanine aminotransferase (ALT), aspartate transaminase (AST), Serum creatinine (Scr) and blood urea nitrogen (BUN) also suggested protective effects of HMIs on liver and kidney function. However, the high heterogeneity observed in several analyses highlights the need for standardized experimental designs and further studies to confirm these findings and to explore their underlying mechanisms. Thus, our meta-analysis indicates that HMIs improve transplantation outcomes in animal models. These results lay a solid foundation for translating HMIs into clinical strategies for improving transplantation outcomes.

**Systematic review registration:**

https://www.crd.york.ac.uk/PROSPERO/view/CRD420251002755, identifier crd420251002755.

## 1 Introduction

In 1954, Peter Bent Brigham Hospital performed the first successful kidney transplantation in human history, marking the beginning of clinical transplantation and heralding one of the most significant achievements in medicine (1). Today, organ transplantation is rapidly developing as a reliable option for patients with end-stage organ failure. Among these, liver, heart, and kidney transplants, as the three major types of solid organ transplantation, have been widely adopted worldwide and have achieved remarkable outcomes. Liver transplantation is the only recognized and widely accepted treatment option for end-stage liver diseases, such as acute fulminant liver failure, hepatocirrhosis, hilar cholangiocarcinoma and hepatocellular carcinoma (2). Heart transplantation offers the optimal survival benefit for patients with end-stage heart failure (3), while kidney transplantation significantly improves the quality of life and reduces mortality rates in patients with renal failure, and is a preferred therapeutic approach (4). Although the survival rate and survival time of transplant recipients are steadily improving (5, 6), there are more postoperative complications, while the quality of life is still poor (7). Recent complications after transplantation are mainly primary graft dysfunction, rejection of an allograft, post-transplant infections and so on. Other long term complications include recurrence of viral hepatitis and autoimmune hepatitis, metabolic syndrome and cardiovascular complications, etc. (8).

The occurrence of the complications is often related to immune-mediated rejection, so an immunosuppressant (IS) is often used after surgery to manage graft rejection and reduce irreversible immune-mediated graft injury (9). The most common regimen is a calmodulin phosphatase inhibitor (CNI, usually tacrolimus), or a CNI containing an antimetabolite (azathioprine or mycophenolate mofetil), with or without corticosteroids (10). However, ISs can also cause side effects, such as tumor recurrence, liver and kidney damage, metabolic syndrome and other related infectious complications, and impose a huge economic burden on transplant patients (11). Some researchers believe that the long-term survival of patients is significantly affected by complications related to excessive immunosuppression (9). Therefore, a delicate balance between effective control of immune-mediated rejection and minimization of the side effects of conventional immunosuppressants has become a critical focus of current research. This has driven efforts to explore alternative or adjunctive immunosuppressive therapies.

Chinese medicine is a traditional Chinese herbal therapy that has been widely used to treat numerous diseases for thousands of years. It has the advantages of low costs with low side effects and has unlimited potential. The ingredients of Chinese medicine are the compounds or chemicals extracted from Chinese herbs. Many recent studies have shown that Chinese herbal medicine ingredients (HMI) exert anti-inflammatory and immunoregulatory effects, particularly for the treatment of autoinflammatory diseases and transplant rejection. For example, kaempferol and berberine can extend transplant survival and promote the induction of immune tolerance to allografts (12, 13), while artemisinin or dihydroartemisinin can ameliorate immune-mediated inflammatory diseases or psoriasis (14, 15). However, the mechanisms underlying the suppression of inflammation and immune-mediated heart, liver, and kidney transplant rejection by HMI remain ambiguous. Thus, we conducted a meta-analysis of inflammatory factors, organ function and other outcomes in animal models of heart, liver, and kidney transplantation. This study systematically explored the potential mechanisms by which HMI modulates organ transplant rejection, and aimed to determine their specific therapeutic efficacy.

## 2 Materials and methods

This study followed the Preferred Reporting Items for Systematic Reviews and Meta-Analyses (PRISMA) guidelines and adhered to the PRISMA checklist (16). We have registered this study with the International Prospective Register of Systematic Reviews (PROSPERO) on 2 March 2025 under registration number CRD420251002755.

### 2.1 Search strategy

We searched the databases, including PubMed, EMBASE, Cochrane Library, Web of Science, Wang Fang, China National Knowledge Infrastructure (CNKI),China Science and Technological Journal Database (VIP), and Chinese Biomedical Literature Database(CBM) from inception to January 1 2025. We used the following terms: ‘organ transplantation’, ‘Chinese medicine’, ‘herbal medicine’ and ‘rodent’. The detailed search strategy can be found in **Supplementary Appendix 1**.

### 2.2 Inclusion criteria

Two researchers (JL and ZW) jointly developed the inclusion and exclusion criteria for this review. The inclusion criteria for the review were as follows (1): Rat or mouse models of heart, liver, and kidney transplantation; (2) animal models of heart, liver, and kidney transplantation without strain, sex and age restrictions that were successfully established in different ways; (3) for the intervention, herbal medicine ingredients were used; (4) the restricted transplantation method was allogeneic heart, liver, and kidney transplantation.

### 2.3 Exclusion criteria

(1) studies that did not establish a suitable animal model, such as other organ transplants, xenotransplants or artificial transplants; (2) studies in which the interventions used in the treatment group were a combination of traditional Chinese medicine, capsules or individual drugs or other therapies;(3) studies that did not meet the inclusion criteria after manual screening were also excluded.

### 2.4 Data extraction

Two researchers (JL and ZW) independently extracted the following data: (1) year of publication and name of the first author; (2) basic information about the experimental animals (e.g. species, strain, age, body weight and number); (3) type of organ transplantation; (4) intervention characteristics, including the type of HMI, duration of administration, administration mode and the processing of the control group; (5) outcome indicators: inflammatory cytokines such as tumor necrosis factor-α (TNF-α), interleukin-1β (IL-1β), interferon-gamma(IFN-γ), interleukin-4 (IL-4), interleukin-10 (IL-10), interleukin-2 (IL-2), interleukin-12 (IL-12); liver function indicators such as ALT, AST, total bilirubin (TBIL); kidney function indicators such as BUN and Scr; oxidative stress markers such as malondialdehyde (MDA) and superoxide dismutase (SOD); apoptosis markers such as apoptosis index (AI), B-cell lymphoma-2 (Bcl-2), Bcl-2/Bax, cysteine-dependent aspartate-specific protease (Caspase)-3; immune cell subsets such as CD4^+^% in splenocytes (SPCs)/lymph node cells (LNCs), CD8^+^% in SPCs/LNCs, CD4^+^/CD8^+^, CD3^+^%, CD4^+^Foxp3^+^ Treg% in SPC, CD11c^+^CD86^+^% in SPCs/LNCs,CD11c^+^CD80^+^% in SPCs/LNCs; Banff schema and MST. When data were presented only graphically, values were estimated from the graphs using GetData Graph Digitizer 2.26.

### 2.5 Risk of bias assessment

Two researchers (JL and ZW) independently assessed the risk of bias using the Systematic Review Center for Laboratory Animal Experimentation (SYRCLE) risk of bias tool (RoBT) (17). The RoBT can assess deviations in the following 10 areas: (1) sequence generation, (2) baseline characteristics, (3) allocation concealment, (4) random housing, (5) blinding of caregivers and investigators, (6) random outcome assessment, (7) blinding of outcome assessment, (8) incomplete outcome data, (9) selective outcome reporting, and (10) other sources of bias. Two researchers negotiated to resolve the dispute over the assessment and, when necessary, a third researcher (HL) was contacted for arbitration.

### 2.6 Statistics

Data analysis was performed using R 4.3 software. All outcomes were treated as continuous variables. When studies reported outcomes using different measures or units, the standardized mean difference (SMD) was used as the effect size index. When studies reported outcomes using same measures or units, mean difference (MD) was used as the effect size index. Confidence intervals (CIs) were set at 95%, and a p-value less than 0.05 was considered statistically significant. Heterogeneity was assessed using the Q-test and the I² statistic. If I² ≤ 50%, a common effect model was used, otherwise a random effect model was utilized. Sensitivity analyses were performed to assess the stability and reliability of the results.

## 3 Results

### 3.1 Literature screening process

**Figure 1:**
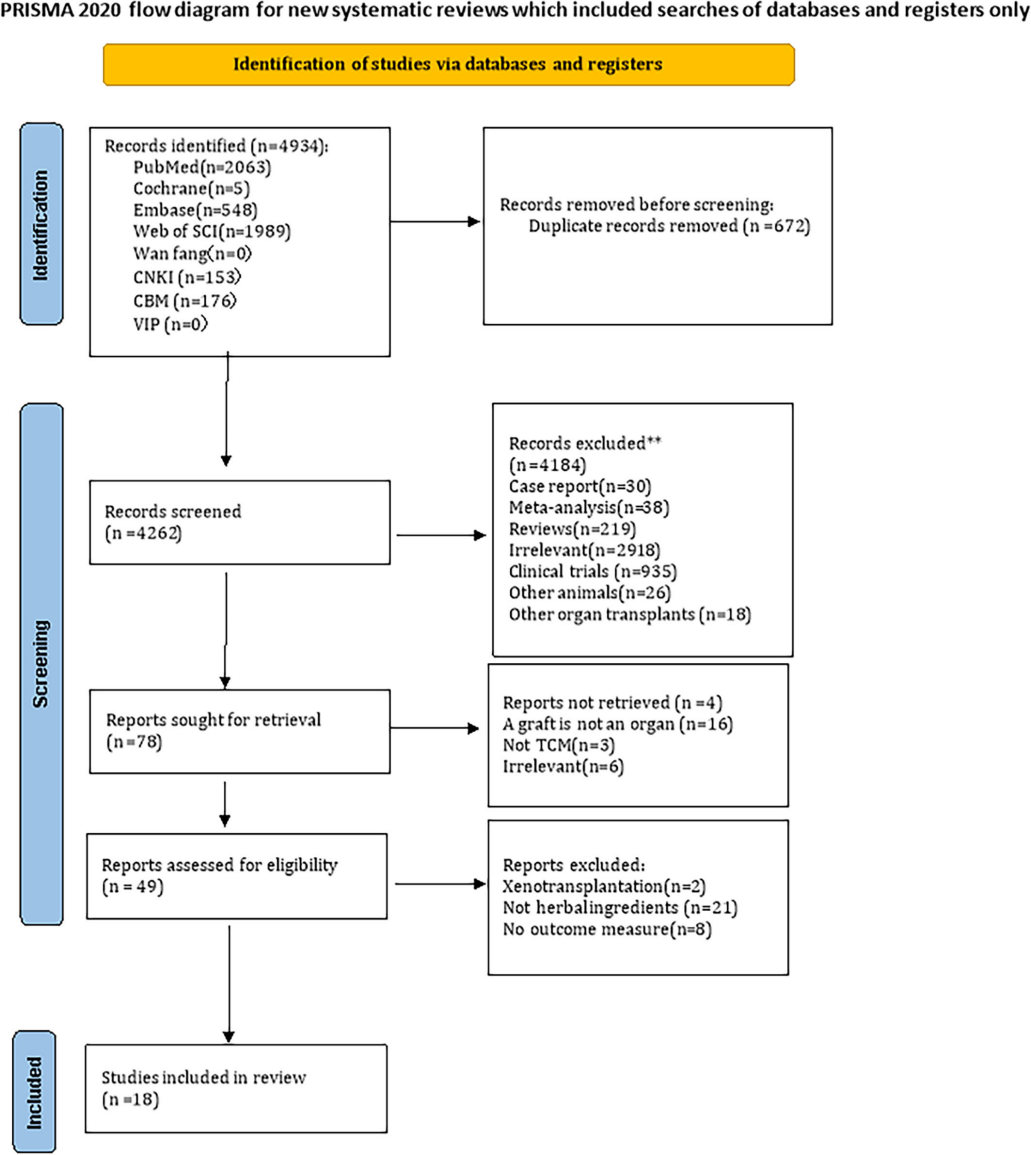
PRISMA flow chart is shown. This study followed the Preferred Reporting Items for Systematic Reviews and Meta-Analyses (PRISMA) guidelines and adhered to the PRISMA checklist.

4934 articles were retrieved from the database according to the search strategy set by two independent investigators (JL and ZW). 672 duplicates were eliminated. First, through reading titles and screening abstracts, other types of articles were eliminated: 219 reviews, 38 Meta analysis, 30 case reports, 935 clinical trials, 26 animals other than rodents, 18 organ transplantation other than liver, heart and kidney, and 2918 unrelated topics. Then, search reports were sought with 4 unsearched reports excluded, 3 articles not associated with traditional herbal medicine, 16 articles concerning non-organ transplantation and 6 irrelevant reports. Next, 49 articles were excluded by reading the full text for the following reasons: (1) Xenotransplantation in 2 articles; (2) Not herbal ingredients in 21 articles; (3) No outcome measure in 8 articles. Finally, 18 articles (18–35) were included in our study (**Figure 1**).

### 3.2 Literature characteristics

18 studies with a total of 357 animals were included, with 319 rats and 38 mice. These included 10 studies (20, 21, 23, 24, 26–30, 32) in English and 8 studies (18, 19, 22, 25, 31, 33–35) in Chinese. These studies were published between 2004 and 2022 (**Supplementary Table 1**). Classification of HMI: emodin (22, 25), artemisinin (34, 35), or berberine(BBR) (24, 30) was used each in 2 studies as interventions. Sodium tanshinone A sulfonate (19), extract of ginkgo biloba leaves(EGb) (33), caffeic acid(CA) (26), radix codonopsis saponins (21), ligustrazine (20), tanshinone IIA (18), ethanol extract of Poria cocos Wolf (EEPCW) (28), asarinin (29), matrine (32), triptolide (23), glycyrrhizic acid (GA) (27) and ethyl acetate extract of hematoxylin(EEH) (31) were used as intervention measures in only one study. Classification of organ transplantation: 8 articles involving liver transplantation (19, 22, 25, 26, 30, 33–35), 3 kidney transplantation (18, 20, 21), and 7 heart transplantation (23, 24, 27–29, 31, 32).

Animal species and modeling methods: All studies used the rats and mice as the species of experimental animals. In the liver transplantation classification, Spraque Dawley rats were used as the donors and recipients for liver transplantation in 3 studies (19, 26, 33), while Spraque Dawley rats and Wistar rats were used as the donors and recipients, respectively, in 3 studies (25, 34, 35). Wistar rats were used as both the donors and recipients for one study (30), while Lewis and Brown Norway rats were used as donors and recipients, respectively, for another study (22). Allogeneic orthotopic liver transplantation was used in all studies to establish rat models of liver transplantation. 1/2 orthotopic liver transplant was used in one study (19), while the whole liver transplantation was performed in the remaining studies. In the kidney transplantation classification, 2 studies used Spraque Dawley rats as donors and recipients (21). The other studies used Fisher 344 rats and Lewis rats as donors and recipients. In one study each, the transplant recipients were bilaterally nephrectomized (21), unilaterally right nephrectomized (20), or unilaterally left nephrectomized (18) to undergo transplantation. In the heart transplantation, Wistar rats and Dawley rats were used as the donors and recipients for transplantation in 3 studies (28, 29, 31). 2 studies used BALB/c mice and C57BL/6 mice as the donors and recipients (24, 32). One study used C57BL/6 mice and BALB/c mice as the donors and recipients (23), and another study used C57BL/6 mice and CBA mice as the donors and recipients (27). 3 studies utilized heterotopic cardiac transplantation (23, 24, 27) (**Supplementary Table 1**).

Outcome measures included inflammatory factors (TNF-α,IFN-γ, IL-4, IL-10,IL-2, IL-1β,IL-12), liver function indicators (AST, ALT, TBIL), kidney function indicators (BUN, Scr), apoptosis markers (AI,Bcl-2,Bcl-2/Bax, caspase-3), oxidative stress markers (SOD, MDA), immune cell subsets (CD4^+^% in SPCs/LNCs, CD8^+^% in SPCs/LNCs, CD4^+^/CD8^+^, CD3^+^%, CD4^+^ Foxp3^+^ Treg% in SPC, CD11c^+^ CD86^+^% in SPCs/LNCs, CD11c^+^CD80^+^% in SPCs/LNCs), and immunological evaluation markers (Banff schema and MST). 9 studies mentioned immunological evaluation markers (20, 22, 23, 25, 28, 29, 31, 34, 35). 7 studies showed inflammatory factors (23, 24, 26, 29, 30, 33, 34). 8 studies mentioned liver and kidney function indicators (18–21, 26, 33–35). 6 studies revealed apoptosis markers (20–22, 25, 26, 31). 4 studies showed oxidative stress markers (18, 20, 21, 30). Finally, 5 studies mentioned immune cell subsets (23, 24, 27, 28, 32) (**Supplementary Table 1**).

### 3.3 Risk of bias

**Figure 2:**
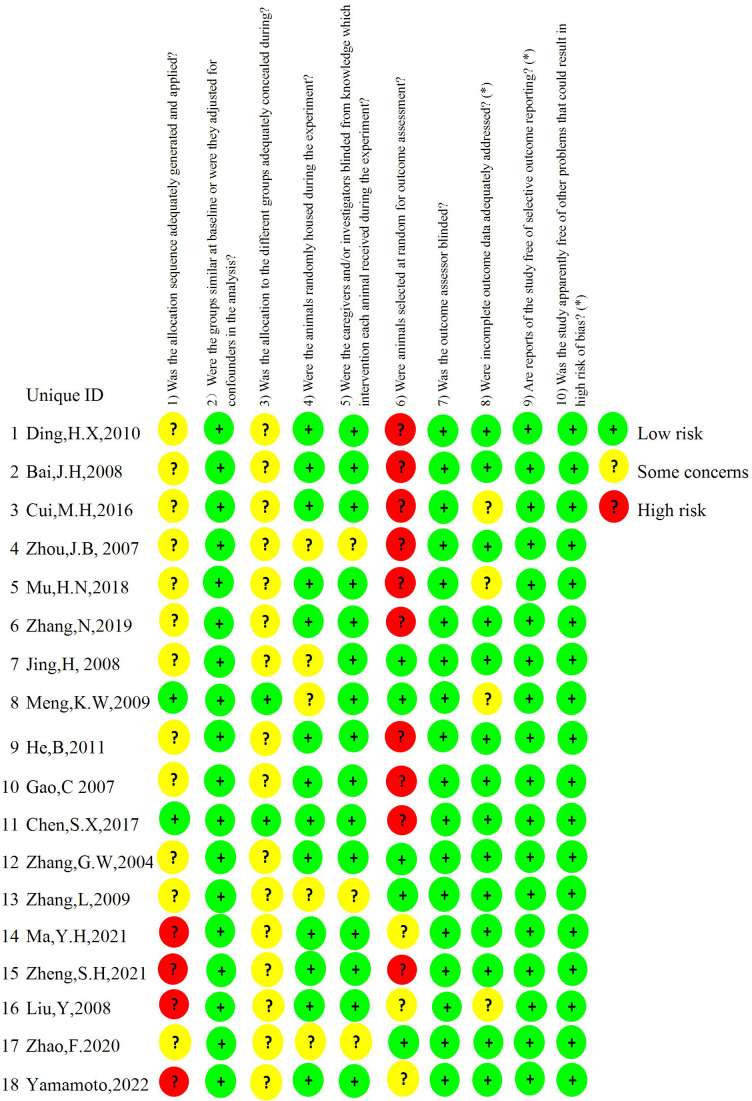
Shown is the risk of bias. The risk of bias is rated in three levels: low risk, some concerns, and high risk. The results were assessed according to the Systematic Review Center for Laboratory Animal Experimentation (SYRCLE) risk of bias tool.

The overall quality of the studies included in the report is moderate. According to the SYRCLE risk of bias tool, the assessment results are as follows (1): 2 studies (18, 25) implemented allocation concealment using random number tables, while 4 studies (23, 24, 27, 32) provided no clear statement regarding randomization procedures. The remaining studies lacked specific descriptions of their randomization methodology. (2) There were no statistically significant differences in baseline data among the animals included in the study. (3) 2 studies (18, 25) adequately implemented allocation concealment using a random number table, while the remaining studies did not clearly specify allocation hiding measures. (4) 13 studies (18–21, 23, 24, 26–28, 30, 32, 34, 35) described the same animal housing conditions while 5 studies (22, 25, 29, 31, 33) did not. (5) 3 studies (29, 31, 33) did not indicate whether the animals were operated and kept in the same environment, while the remaining 15 studies explicitly employed blinding in the same setting. (6) Of the 18 studies that evaluated outcomes, 5 studies (22, 25, 28, 29, 31) randomly selected animals for outcome evaluation, with 10 studies (18–21, 26, 30, 32–35)measuring outcome indicators for all animals within the group, while the method of animal selection for outcome assessment was unclear for the remaining 3 studies (23, 24, 27). (7) 3 studies (24, 27, 30) explicitly indicated that outcome assessments were conducted by personnel independent from the experimental operators, implying that outcome assessor blinding was achieved), and 5 studies (22, 25, 28, 29, 31) randomly selected animals for outcome evaluation. In the remaining studies, all animals in each group were tested or identical measurement protocols were implemented, thus ensuring unaffected outcome measurements. Consequently, all included studies were rated as low risk of bias for this domain. (8) 4 studies (19, 23, 25, 26) failed to evaluate the results of all animals due to death caused by organ transplantation when the outcome indicators were measured, but it was not stated whether the missing data affected the results. (9) All animals in other studies were included in the outcome analysis (10). The expected results of all studies have been reported and there are no other sources of bias (**Figure 2**).

### 3.4 Meta-analysis of immunological evaluation markers

**Figure 3:**
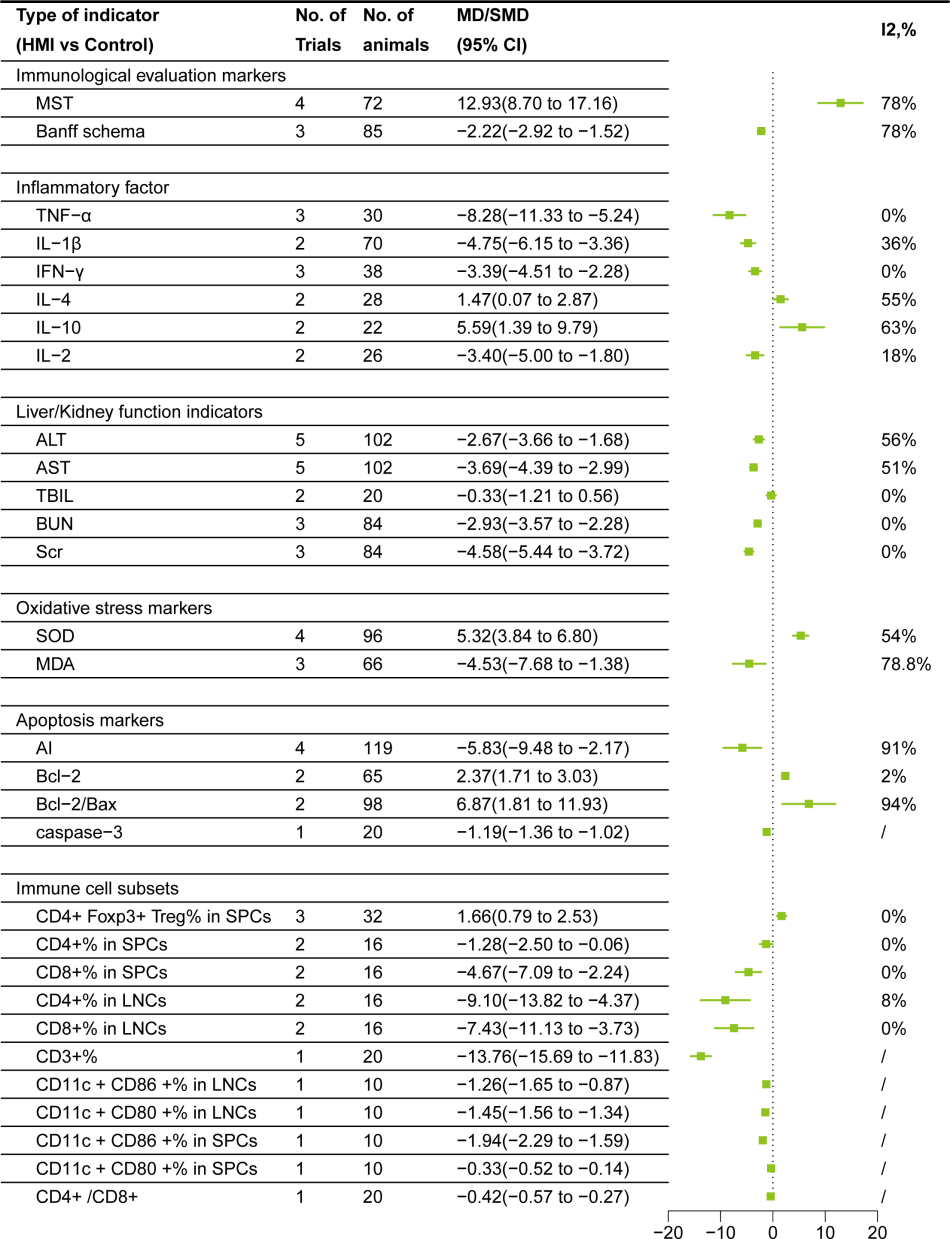
Forest plots of results of meta-analysis.

After sensitivity analysis, I^2^ = 78% for MST after removing Meng KW (25). Random effect model is used in both cases. Data from 8 studies, involving 157 rodents, compared HMI and control group:1. Banff Schema:The Banff Schema score(MD= -2.22, 95% CI= -2.92, -1.52)of HMI group was lower than that of control group; 2. MST:MST in HMI group was significantly prolonged(MD = 12.93, 95% CI =8.70,17.16) (**Figure 3**).

### 3.5 Meta-analysis of inflammatory factors

After sensitivity analysis, I^2^ = 0% for TNF-α after removing Mu HN (26), and I^2^ = 36% for IL-1β after removing Zhou JB (33). I^2^ = 55% for IL-4 after removing Bai JH (34), and I^2^ = 63% for IL-10 after removing Zhang L (29). Random effect model is used in all cases. Data from a total of 232 rodents, comparing the HMI with control group, showed that: 1. Pro-inflammatory factors: TNF-α (SMD= -8.28, 95%CI = -11.33, -5.24), IL-1β (SMD= -4.75, 95%CI = -6.15, -3.36), IFN-γ (SMD= -3.39, 95%CI = -4.51, -2.28), and IL-2 (SMD= -3.40, 95%CI = -5.00,-1.80)were lower than those in the control group after HMI treatment. 2. Anti-inflammatory factors: IL-4 (SMD=1.47, 95%CI =0.07, 2.87), IL-10 (SMD=5.59, 95%CI =1.39, 9.79) were higher than those in the control group. These results suggest that HMIs can improve post-transplant rejection by reducing the contents of pro-inflammatory factors and increasing anti-inflammatory factors (**Figure 3**).

### 3.6 Meta-analysis of liver and kidney function indicators

After sensitivity analysis, the results for ALT and AST after removing Cui MH (19) were I^2^ = 56% and I^2^ = 51%, respectively. Random effects model is used in all cases. Comparing HMI with the control group, the results showed that: 1. Liver function: The TBIL level decreased (SMD = -0.33) in the HMI group, suggesting that HMI may help reduce liver damage and improve bilirubin metabolism. However, since 95% CI was 0, the difference was not statistically significant, and further studies are needed to confirm this effect. Besides, HMI significantly decreased the AST (SMD =-3.69, 95% CI=-4.39, -2.99) and ALT (SMD = -2.67, 95% CI= -3.66, -1.68) of the transplanted animal model. 2. Kidney function: BUN (SMD = -2.93, 95% CI = -3.57, -2.28) and Scr (SMD = -4.58, 95% CI = -5.44, -3.72) decreased in transplant recipients treated with HMI. These results indicate that HMI has a significant benefit on liver and kidney function (**Figure 3**).

### 3.7 Oxidative stress markers meta-analysis

Using random effects model, data from 7 studies involving 66-96 rodents showed the following changes: HMI decreased MDA level(SMD = -4.53, 95% CI = -7.68, -1.38) and increased SOD activity(SMD =5.32, 95% CI = 3.84,6.80). These results suggest that HMI mitigated oxidative damage and promoted graft protection by modulating antioxidant molecules (**Figure 3**).

### 3.8 Meta-analysis of apoptosis markers

After sensitivity analysis, the result for Bcl-2 after removing He B (21) was I^2^ = 2%. Random effects model was used in all cases. Data from 9 studies involving 20-119 rodents showed the following changes: HMI decreased AI(SMD = -5.83,95% CI = -9.48,-2.17)and caspase-3(MD = -1.19, 95% CI = -1.36,-1.02), increased Bcl-2(SMD =2.37, 95% CI = 1.71, 3.03) and Bcl-2/Bax(SMD =6.87, 95% CI = 1.81,11.93) ratio, indicating that HMI promotes graft protection by inhibiting graft apoptosis (**Figure 3**).

### 3.9 Meta-analysis of immune cell subsets

CD4^+^Foxp3^+^ Treg% in SPCs adopted a common effect model since its I^2^ = 0%, and the rest used random effect model. Data from 176 rodents showed the following changes: HMI decreased CD3^+^%(MD = -13.76, 95% CI = -15.69, -11.83), CD4^+^/CD8^+^ ratio(MD = -0.42, 95% CI = -0.57,-0.27), CD11c ^+^CD86 ^+^%(MD = -1.26, 95% CI = -1.65,-0.87), CD11c ^+^CD80^+^% (MD = -1.45, 95% CI = -1.56, -1.34) in LNCs and CD11c^+^CD86^+^% (MD = -1.94, 95% CI = -2.29, -1.59) CD11c^+^CD80^+^%(MD = -0.33, 95% CI = -0.52,-0.14) in SPCs. Additionally, HMI reduced CD4^+^%(SMD = -1.28, 95% CI = -2.50, -0.06), CD8^+^%(SMD = -4.67, 95% CI = -7.09, -2.24) in SPCs, and CD4^+^%(SMD = -9.10, 95% CI = -13.82, -4.37), CD8^+^%(SMD = -7.43, 95% CI = -11.13, -3.73) in LNCs. At the same time, HMI increased CD4^+^Foxp3^+^ Treg% in SPCs (SMD = 1.66, 95% CI =0.79,2.53) (**Figure 3**).

### 3.10 Subgroup meta-analysis

#### 3.10.1 Egb

**Figure 4:**
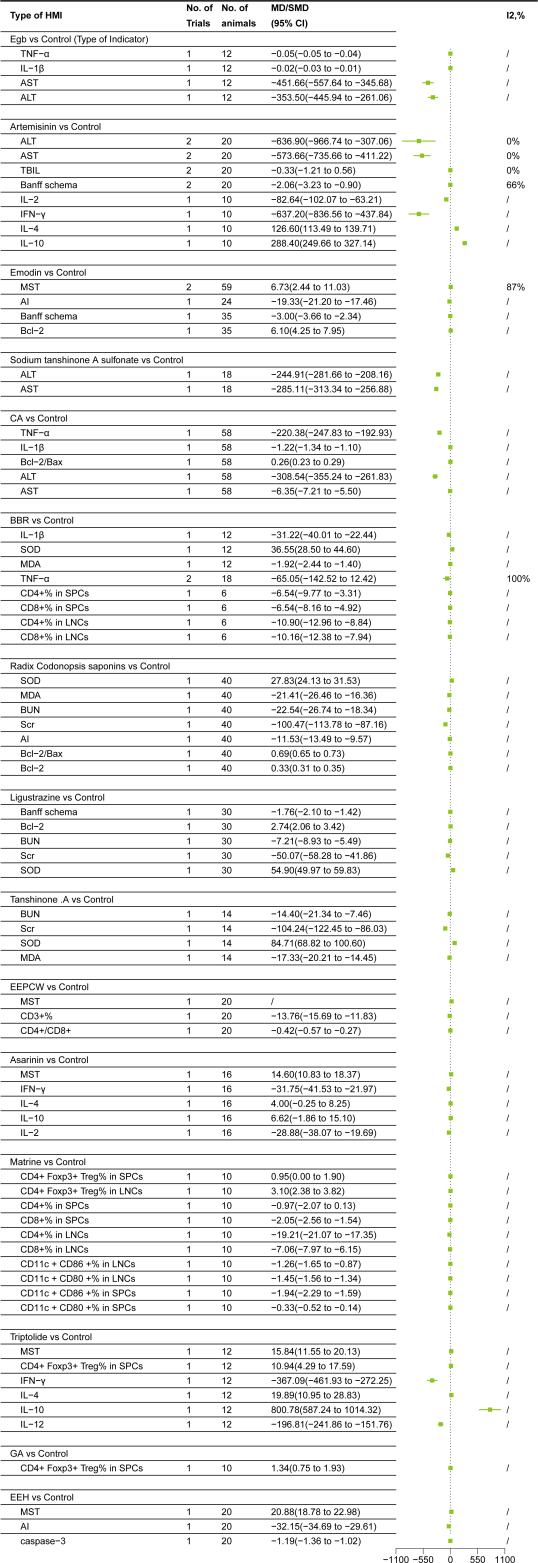
Forest plots of results of subgroup meta-analysis.

Egb subgroup meta-analysis based on HMI classification showed the following changes (33): 1. Inflammatory factors: Egb decreased IL-1β(MD = -0.02, 95% CI = -0.03, -0.01)and TNF-α (MD = -0.05, 95% CI = -0.05, -0.04); 2. Liver function: Egb reduced AST(MD = -451.66, 95% CI = -557.64, -345.68)and ALT(MD = -353.50, 95% CI = -445.94,-261.06)levels (**Figure 4** **).**

#### 3.10.2 Artemisinin

Artemisinin subgroup meta-analysis based on HMI classification showed the following changes (34, 35):1. Inflammatory factors:Artemisinin decreased IL-2(MD = -82.64, 95% CI = -102.07, -63.21)and IFN-γ (MD = -637.20, 95% CI= -836.56, -437.84), increased IL-4(MD = 126.60, 95% CI = 113.49, 139.71), IL-10(MD = 288.40, 95% CI=249.66, 327.14); 2. Liver function:Artemisinin decreased TBIL(SMD = -0.33, 95% CI = -1.21,0.56), ALT(MD = -636.90,95% CI = -966.74, -307.06), AST(MD = -573.66, 95% CI = -735.66, -411.22)levels. TBIL, however, was not statistically significant (95% CI included 0), warranting further research to verify the effect; 3. Immunological evaluation markers: Artemisinin decreased Banff schema score (MD = -2.06, 95% CI= -3.23, -0.90) (**Figure 4** **).**

#### 3.10.3 Emodin

Emodin subgroup meta-analysis based on HMI classification showed the following changes (22, 25):1. Apoptosis markers:Emodin increased Bcl-2(MD = 6.10, 95% CI =4.25,7.95), but decreased AI(MD = -19.33, 95% CI = -21.20,- 17.46); 2. Immunological evaluation markers: Emodin reduced Banff schema score (MD = -3.00, 95% CI= -3.66,- 2.34), and prolonged MST(MD = 6.73, 95% CI = 2.44, 11.03) (**Figure 4** **).**

#### 3.10.4 Sodium tanshinone A sulfonate

Sodium tanshinone A sulfonate subgroup meta-analysis based on HMI classification showed the following changes (19):Sodium tanshinone A sulfonate decreased AST(MD = -285.11, 95% CI = -313.34, -256.88)and ALT(MD = -244.91, 95% CI = -281.66, -208.16)levels (**Figure 4** **).**

#### 3.10.5 CA

CA subgroup meta-analysis based on HMI classification showed the following changes (26):1. Apoptosis markers:CA increased Bcl-2/Bax ratio(MD = 0.26, 95% CI =0.23,0.29); 2. Inflammatory factors:CA decreased IL-1β(MD = -1.22, 95% CI = -1.34,-1.10)and TNF-α (MD= -220.38, 95% CI = -247.83, -192.93); 3. Liver function:CA reduced AST(MD = -6.35, 95% CI = -7.21,-5.50)and ALT(MD = -308.54, 95% CI = -355.24, -261.83)levels (**Figure 4**).

#### 3.10.6 BBR

BBR subgroup meta-analysis based on HMI classification showed the following changes (24, 30):1. Oxidative stress markers:BBR increased SOD(MD = 36.55, 95% CI =28.50, 44.60) and decreased MDA(MD = -1.92, 95% CI =-2.44, -1.40); 2. Inflammatory factors:BBR decreased IL-1β(MD = -31.22, 95% CI = -40.01,-22.44) and TNF-α (MD= -65.05, 95% CI = -142.52,12.42). TNF-α, however, was not statistically significant (95% CI included 0), warranting further research to verify the effect; 3. Immune cell subsets: BBR reduced CD4^+^%(MD = -6.54, 95% CI = -9.77, -3.31), CD8^+^%(MD = -6.54, 95% CI = -8.16,-4.92) in SPCs, and CD4^+^%(MD = -10.90, 95% CI = -12.96, -8.84) and CD8^+^%(MD = -10.16, 95% CI = -12.38, -7.94) in LNCs (**Figure 4**).

#### 3.10.7 Radix codonopsis saponins

Radix codonopsis saponins subgroup meta-analysis based on HMI classification showed the following changes (21):1. Oxidative stress markers:Radix codonopsis saponins increased SOD(MD = 27.83, 95% CI =24.13, 31.53) and decreased MDA(MD = -21.41, 95% CI = -26.46, -16.36); 2.Apopotosis markers:Radix codonopsis saponins increased Bcl-2(MD =0.33, 95% CI =0.31,0.35) and Bcl-2/Bax (MD=0.69, 95% CI =0.65, 0.73) and reduced AI(MD = -11.53, 95% CI = -13.49, -9.57); 3. Kidney function: It decreased Scr(MD = -100.47, 95% CI = -113.78, -87.16) and BUN(MD = -22.54, 95% CI= -26.74, -18.34) levels (**Figure 4**).

#### 3.10.8 Ligustrazine

Ligustrazine subgroup meta-analysis showed the following changes (20): 1. Oxidative stress markers:Ligustrazine increased SOD(MD = 54.90, 95% CI=49.97, 59.83); 2.Apopotosis markers:Ligustrazine increased Bcl-2(MD =2.74, 95% CI=2.06, 3.42); 3.Kidney function:Ligustrazine decreased Scr(MD = -50.07, 95% CI = -58.28, -41.86) and BUN(MD = -7.21, 95% CI=- 8.93, -5.49) levels; 4. Immunological evaluation markers: Ligustrazine reduced Banff schema score (MD = -1.76, 95% CI = -2.10, -1.42) (**Figure 4** **).**

#### 3.10.9 Tanshinone IIA

TanshinoneIIA subgroup meta-analysis showed the following changes (18):1. Oxidative stress markers:Tanshinone IIA increased SOD(MD = 84.71, 95% CI =68.82,100.60) and decreased MDA(MD = -17.33, 95% CI =-20.21, -14.45); 2.Kidney function:It decreased Scr(MD = -104.24, 95% CI = -122.45, -86.03) and BUN(MD = -14.40, 95% CI= -21.34, -7.46) levels (**Figure 4** **).**

#### 3.10.10 EEPCW

EEPCW subgroup meta-analysis based on HMI classification showed the following changes (28):1. Immunological evaluation markers: EEPCW prolonged MST(MD = - 23.3)2. Immune cell subsets: EEPCW decreased CD3^+^%(MD = -13.76, 95% CI = -15.69, -11.83) and CD4^+^/CD8^+^ ratio(MD = -0.42, 95% CI = -0.57, -0.27**) (** **Figure 4** **).**

#### 3.10.11 Asarinin

Asarinin subgroup meta-analysis showed the following changes (29):1. Immunological evaluation markers: Asarinin prolonged MST(MD = 14.60, 95% CI =10.83, 18.37)2.Inflammatory factors: Asarinin decreased IL-2(MD = -28.88, 95% CI = -38.07, -19.69)and IFN-γ (MD = -31.75, 95% CI= -41.53, -21.97), increased IL-4(MD = 4.00, 95% CI = -0.25,8.25)and IL-10(MD = 6.62, 95% CI= -1.86, 15.10). However, since the 95% CI of IL-4 and IL-10 included 0, the difference was not statistically significant, and further studies are needed to confirm this effect (**Figure 4**).

#### 3.10.12 Matrine

Matrine subgroup meta-analysis showed the following changes (32):It increased CD4^+^ Foxp3^+^Treg% in SPCs (MD = 0.95, 95% CI =0.00,1.90)and in LNCs (MD = 3.10, 95% CI =2.38, 3.82), and decreased CD4^+^%(MD = -0.97, 95% CI = -2.07, 0.13), CD8^+^%(MD =-2.05, 95% CI = -2.56,-1.54) in SPCs; CD4^+^%(MD =-19.21,95% CI =-21.07,-17.35) and CD8^+^%(MD = -7.06, 95% CI = -7.97, -6.15) in LNCs; CD11c^+^ CD86 ^+^%(MD = -1.26, 95% CI = -1.65, -0.87) and CD11c ^+^CD80 ^+^%(MD = -1.45, 95% CI = -1.56, -1.34) in LNCs; CD11c ^+^CD86 ^+^%(MD = -1.94, 95% CI = -2.29, -1.59) and CD11c ^+^CD80 ^+^%(MD = -0.33, 95% CI = -0.52, -0.14) in SPCs. CD4^+^% or CD4^+^Foxp3^+^ Treg% in SPCs, however, was not statistically significant (95% CI included 0), warranting further research to verify the effect (**Figure 4** **).**

#### 3.10.13 Triptolide

Triptolide subgroup meta-analysis showed the following changes (23): 1. Immunological evaluation markers: Triptolide prolonged MST(MD = 15.84, 95% CI =11.55, 20.13). 2. Inflammatory factors: Triptolide decreased IL-12(MD = -196.81, 95% CI = -241.86, -151.76) and IFN-γ (MD = -367.09, 95% CI= -461.93, -272.25), increased IL-4(MD =19.89, 95% CI =10.95, 28.83) and IL-10(MD = 800.78, 95% CI=587.24,1014.32). 3. Immune cell subsets:Triptolide increased CD4^+^Foxp3^+^ Treg% in SPCs (MD = 10.94, 95% CI =4.29, 17.59) (**Figure 4** **).**

#### 3.10.14 GA

GA subgroup meta-analysis (27) showed that it increased CD4^+^Foxp3^+^ Treg% in SPCs (MD = 1.34, 95% CI =0.75, 1.93) (**Figure 4** **).**

#### 3.10.15 EEH

EEH subgroup meta-analysis showed the following changes (31): 1. Immunological evaluation markers: EEH prolonged MST(MD = 20.88, 95% CI =18.78, 22.98); 2. Apoptosis markers:emodin reduced caspase-3(MD = -1.19, 95% CI = -1.36, -1.02) and AI(MD = -32.15, 95% CI = -34.69, -29.61) (**Figure 4** **).**

## 4 Discussion

Previous research on the treatment of post-transplantation complications and graft rejection primarily focuses on immunosuppressants, including calcineurin inhibitor (CNI), corticosteroid and mammalian target of rapamycin (mTOR) inhibitor (36). Recent studies have suggested that HMI may serve as a promising alternative therapy for enhancing immunosuppression and improving transplantation outcomes. Many animal studies have shown that HMI can induce immune tolerance to allografts (12, 13). However, no studies have systematically analyzed the effects of HMI on inflammatory cytokines, oxidative stress index, apoptosis index, immunophenotyping indicators and liver or kidney injury/function in organ transplantation settings. This preclinical systematic review and meta-analysis included 18 studies with a total of 357 experimental animals to assess whether HMI can attenuate inflammation and improve organ function in animal models, providing further insights into the impacts of HMIs on transplant survival, post-transplantation complications, and their underlying mechanisms of action. Given that HMIs are derived from natural products and may be less toxic if the appropriate doses with high purity are used, and that conventional immunosuppressive agents have considerable side effects, they exhibit a significant potential for clinical translation. Thus, it is compelling to conduct clinical trials using HMIs, at least as a complementary measure, in transplanted patients at the near future.

Immune-mediated transplant rejection, a main problem with transplanted patients, is highly complex. The related outcomes following organ transplantation include acute cellular rejection, graft-versus-host disease (GVHD), and recurrence of pre-transplantation autoimmune diseases (37). Generally, immune-mediated rejection can be managed by depleting lymphocytes, blocking their signaling pathways, suppressing their activation, and neutralizing alloantibodies (38). Upon antigen stimulation, CD4^+^ T cells differentiate into Th1 and Th2 subsets. Th1 cells secrete IL-2, interferon-γ (IFN-γ), and IL-12, while Th2 cells produce IL-4 and IL-10 (39, 40). Studies suggest that elevated levels of IL-4 and IL-10 indicate immune acceptance (41). Activated mast cells play a central role in initiating and sustaining inflammation by producing TNF-α (42). IL-1β and IL-12 are other common pro-inflammatory factors. Those inflammatory factors once released, intracellular signaling pathways will be activated, such as interleukin-1 receptor-associated kinase 4 (IRAK4) and NF-κB signaling pathways, ultimately leading to further expression of pro-inflammatory cytokines and inflammasome proteins (43). TNF-α production is a hallmark of acute rejection, while IL-1β synergizes with TNF-α in more severe rejection responses (44).

On the other hand, apoptosis, a significant manifestation of cellular damage during organ transplant rejection, shows AI that correlates with the severity of rejection. The Bcl-2 family, such as Bcl-2, is an anti-apoptotic protein that protects hepatocytes from undergoing apoptosis (45). In T cell-mediated rejection, naive CD8^+^ T lymphocytes recognize alloantigens and costimulatory signals and induce parenchymal cell apoptosis in the graft via the Fas cell surface death receptor - Fas ligand (Fas-FasL) pathway, which produces pro-inflammatory cytokines that in turn attract neutrophils and/or monocyte-macrophages to induce further graft damage (46). Caspase-3, a frequently activated death protease and a key member of the caspase family, serves as a critical mediator of apoptosis by catalyzing the specific cleavage of numerous essential cellular proteins (47). Additionally, TNF-α, IFN-γ and parenchymal cell apoptosis are experimentally confirmed to be closely associated with notable increases in acute allograft rejection (48).

Previous studies have shown that the ratio of AST to ALT serves as a marker of liver injury (49), with elevated ALT and AST levels potentially indicating liver damage, fatty liver disease, and/or oxidative stress (50). TBIL, a byproduct of heme breakdown, is crucial for diagnosing liver diseases through measurement of plasma bilirubin (51). AST, ALT, and TBIL are sensitive indicators of liver tissue damage and often increase with acute rejection, thus serving as effective markers for monitoring liver transplant rejection (52). The kidneys are primarily responsible for excreting metabolic waste products and their health status is crucial for overall physiological function. Scr is an end product of muscle metabolism, released into the bloodstream and continuously cleared by the kidneys. Failure to clear Scr indicates severe renal dysfunction (53). BUN represents the nitrogen content in urea, which is filtered from the blood by healthy kidneys. When kidney function is impaired, BUN levels rise (54). Therefore, Scr is often evaluated in conjunction with BUN level to determine renal function. Banff schema is a standardized scale for identifying, naming, and grading acute allograft rejection and is widely adopted by scientific journals as an internationally recognized scoring system (55).

Oxidative stress is induced by an imbalance between the production of free radicals/peroxides and the body’s antioxidant system (56). MDA, a marker of oxidative stress, is a highly oxidized product generated under oxidative stress conditions. SOD, the most important free radical scavenger in the body, can reduce the extent of inflammatory responses and mitigate lipid peroxidation. During oxidative stress, SOD is consumed (57).

DCs are professional antigen-presenting cells that bridge innate and adaptive immunity, playing a critical role in initiating T cell-dependent immune responses. DCs exist in an immature state in tissue. Upon activation by inflammatory stimuli or inflammatory factors, DCs undergo a maturation process characterized by upregulation of co-stimulatory molecules (CD40, CD80, CD86) and adhesion molecules (CD54, CD58), and migrate to lymphoid organs to activate naive and memory lymphocytes (32). Organ transplantation triggers a T-cell-mediated immune response, with CD4^+^ T cells producing pro-inflammatory cytokines and assisting in CD8^+^ T cell responses (58). Therefore, the reduction of CD3^+^, CD4^+^, and CD8^+^ T cells is conducive to the protection of allografts. On the other hand, Tregs can delay or prevent rejection. Although Tregs expressing CD4^+^CD25^+^Foxp3^+^ only account for 2%-5% of circulating T cells, they are essential for maintaining immune homeostasis and inducing immune tolerance (59).

Our study showed that HMIs significantly prolonged MST and lowered Banff schema scores, thus reducing acute rejection. Their mechanisms of action are manifested in various synergistic effects. In terms of immune regulation, HMIs decreased the expression of pro-inflammatory factors (TNF-α, IL-1β, IFN-γ, IL-2, IL-12) and promoted the expression of anti-inflammatory factors (IL-10, IL-4). In terms of antioxidant and anti-apoptotic effects, HMIs significantly reduced MDA level, enhanced SOD activity, upregulated the Bcl-2/Bax ratio and Bcl-2 expression, and suppressed caspase-3 expression and AI. HMIs could also inhibit DC maturation and T cell proliferation by decreasing the percentage of CD3^+^, CD8^+^, CD4^+^, CD11c ^+^CD80 ^+^, or CD11c ^+^CD86 ^+^ cells in transplant recipients, but increasing the proportion of CD4^+^Foxp3^+^ Treg cells. Additionally, HMI improved liver and kidney function in transplant animals, as evidenced by a significant reduction in AST, ALT, BUN and Scr levels. Although individual studies suggested that HMIs improved TBIL level, the pooled meta-analyses demonstrated that the confidence interval crossed the null line, indicating that this effect was not statistically significant. This could be attributed to limited sample sizes, high heterogeneity among studies, or simply insufficient therapeutic efficacy of HMIs on TBIL.

Subgroup analyses revealed that HMIs exerted immunomodulatory effects on transplant rejection by regulating liver and kidney function, ameliorating oxidative stress, inhibiting apoptosis, and modulating inflammatory cytokines. However, the reliability of these findings requires further validation, as the majority of current evidence on HMIs is derived from a single study. Future research should focus on the following priorities (1): expanding experimental validation of bioactive compounds, particularly those supported by only a single study, through independent replications across multiple models; (2) elucidating the precise targets and pathways of HMIs by integrating network pharmacology and multi-omics approaches. These efforts will help distinguish stochastic observations from genuine biological effects and provide a solid foundation for the translational application of HMIs.

**Figure 5:**
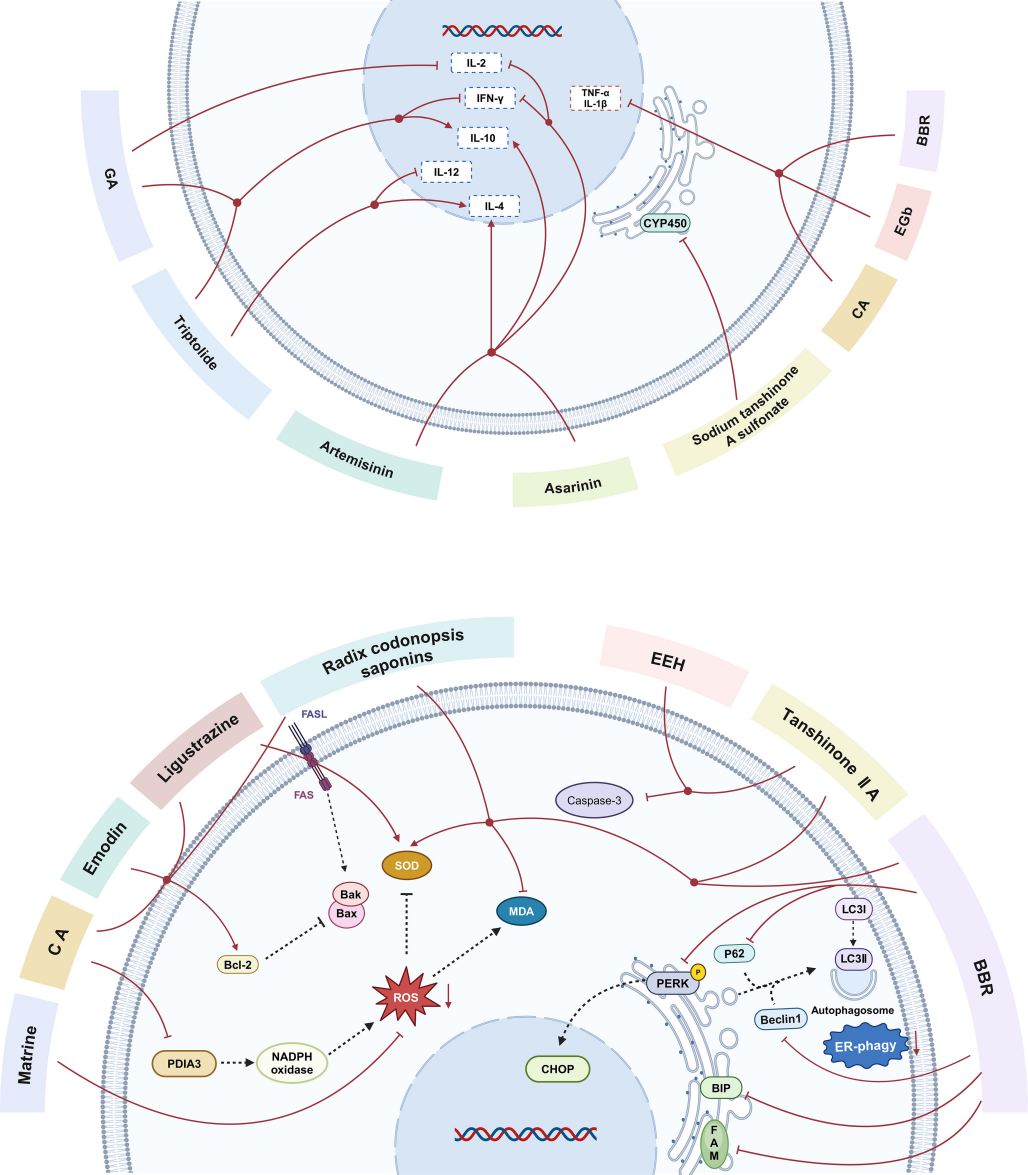
The main mechanisms by which herbal medicine ingredients(HMIs)regulate immune-mediated rejection in animal models of transplantation. CA, caffeic acid; BBR, berberine; EGb, extract of ginkgo biloba leaves; GA, glycyrrhizic acid; EEH, ethyl acetate extract of hematoxylin; FASL,factor-related Apoptosis ligand; FAS, Fas cell surface death receptor Gene; MDA,Malondialdehyde; SOD,Superoxide dismutase;Bcl-2, B-cell lymphoma-2; Bax, Bcl-2-Associated X; Bak, Bcl-2 Antagonist Killer; PDIA3, protein disulfide isomerase family A member 3; NADPH, nicotinamide adenine dinucleotide phosphate; ROS, reactive oxygen species; CYP450, cytochrome P450; PERK, protein kinase R-like endoplasmic reticulum kinase; p62, sequestosome 1; CHOP, C/EBP-homologous protein; Beclin1, autophagic protein Beclin1; LC3I, Microtubule-Associated Protein 1 Light Chain 3 I; LC3II, Microtubule-Associated Protein 1 Light Chain 3 II;BIP, heavy-chain binding protein; FAM134B,family with sequence similarity 134, member B; ER phagy, endoplasmic reticulum phagy; IL-2, interleukin-2; IL-10, interleukin-10; IL-4, interleukin-4; IFN-γ, Interferon-γ; TNF-α, tumor necrosis factor-α; IL-1, interleukin-1; IL-12, interleukin-1.

Results from our systematic review suggest that HMI may reduce post-transplantation complications and acute organ rejection through the following mechanisms (**Figure 5**):

(1) Improving ischemia reperfusion (I/R) injury and oxidative stress

Ischemia-reperfusion (I/R) injury is a critical issue in organ transplantation and plays a key role in determining both early clinical outcomes and long-term survival. It has been suggested that the core of I/R injury is inflammation, which is closely linked to the generation of reactive oxygen species (ROS) and the activation of Kupffer cells (KCs) (60). After I/R, the excessive production of ROS not only damages cell and organelle membranes, exceeding the body’s clearance capacity, leading to tissue injury, cell apoptosis, and lipid peroxidation, but also promotes the differentiation and maturation of dendritic cells(DC), exacerbating inflammatory responses and further aggravating rejection (21, 32). Zheng et al. have shown that matrine, as an antioxidant, can effectively reduce ROS generation in DCs and inhibit DC maturation by activating the ROS/ERK1/2/NF-κB pathway, thereby alleviating acute rejection (32). Studies by Gao and He et al. have shown that ligustrazine, EEH, and Radix codonopsis saponins protect grafts from ischemia-reperfusion injury through mechanisms involving ROS clearance, reduced MDA production, elevated SOD level, and suppression of lipid peroxidation and inflammatory responses (20, 21, 31). Hepatic KCs are a largest macrophage population in the body, and upon reperfusion injury, they release inflammatory mediators and cytokines, such as TNF-α and IL-1 (61). Zhou et al. demonstrated significant improvement in hepatic morphological changes in the EGb-treated group, with lower levels of TNF-α and IL-1 in the liver tissue compared to the control group. These findings suggest that EGb may have a protective effect against I/R injury, possibly through the inhibition of KC activation, reduction in release of pro-inflammatory cytokines, and subsequent decrease in ROS generation and lipid peroxidation, thus alleviating I/R injury (33). Additionally, Mu et al. observed that in I/R injury, CA treatment significantly reduced protein disulfide isomerase family A member 3 (PDIA3) expression and decreased nicotinamide adenine dinucleotide phosphate (NADPH) oxidase activity. CA treatment protected against oxidative damage by interfering with NADPH oxidase activation, thereby reducing the production of ROS in the graft tissue and improving tissue preservation, microcirculation, and I/R injury (26).

(2) Inhibiting graft cell apoptosis

When immune-mediated rejection occurs after organ transplantation, cytotoxic T lymphocytes (CTLs) are activated and bind to the Fas receptor on graft cells through their expression of FasL, triggering a series of signaling reactions that lead to cell apoptosis (62). According to the staining images and statistical comparison of AI reported by Jing et al., the use of HMI effectively suppressed graft cell apoptosis after organ transplantation (21, 22, 25, 31). The activation of the pro-apoptotic factor Bax leads to the opening of pores on the mitochondrial membrane, increasing membrane permeability and resulting in apoptosis (63). Additionally, Bax can form homodimers to accelerate cell death or heterodimers with Bcl-2 to inhibit cell death (64). Therefore, increasing the Bcl-2/Bax ratio can suppress apoptosis. Researches indicate that HMI can upregulate the expression of Bcl-2 (20, 21, 25),reduce the Bcl-2/Bax ratio (21, 26), and decrease the expression of caspase-3 (31), suggesting that HMI improves graft survival by inhibiting apoptosis.

(3) Suppressing the adaptive and innate immunity

Mature DCs play a central role in initiating, regulating, and sustaining immune responses by activating naive T cells (65). Th1 cells assist in activating cytotoxic CD8^+^ T cells by secreting pro-inflammatory cytokines, such as IL-2 and IFN-γ, and promote pro-inflammatory responses that lead to graft damage (24, 66). Studies have shown that HMIs inhibit cellular immunity through multiple pathways:

(A) Decreasing Th1/Th2 ratio and causing immune deviation: IL-4 inhibits the production of Th1 cytokines, such as IL-2 and IFN-γ, while promoting the clonal expansion of Th2 cells and driving the shift from Th1 to Th2, thereby inducing the immune tolerance (67). IL-10 also suppresses the proliferation of Th1 cells and the synthesis of Th1 cytokines, such as IL-2 and IFN-γ (68). Bai and Liu et al. found that after antigen-presenting cells presented antigens to naive T cells, artemisinin increased the secretion of IL-4 and IL-10 by activated T cells and promoted the differentiation of Th0 cells towards Th2, which led to a decrease in IL-2 and IFN-γ production and inhibition of Th1 cell activation, thus driving the immune responsiveness toward humoral immunity (23, 34). Zhang et al. observed a reduction in the CD4^+^/CD8^+^ ratio, indicating that EEPCW can inhibit CD4^+^ T cell proliferation, thereby suppressing cell-mediated immunity after transplantation (28).

(B) Inhibiting effector T cell proliferation: Ma et al. demonstrated that BBR significantly inhibited the proliferation of CD4^+^ and CD8^+^ T cells and blocked IFN-γ production in CD3^+^ T cells, thereby preventing graft rejection by impairing Th1 cell function (24). Zhang et al. further confirmed that the ethanol extract of Poria cocos wolf reduced the percentage of CD3^+^ T cells and inhibited their proliferation (28).

(C) Inhibiting the maturation of DC: Zheng et al. showed that matrine inhibited DC maturation post-transplantation through the ROS/ERK1/2/NF-κB pathway both *in vitro* and *in vivo (* 32).

(4) Promoting regulatory T cells (Treg)-mediated immunoregulation

Immune regulation is considered one of the key mechanisms for inducing and maintaining transplant tolerance. Among these mechanisms, CD4^+^CD25^+^Foxp3^+^ Tregs promote immune tolerance to an allograft by actively suppressing effector T cell activation, modulating dendritic cell function, inhibiting cytokine release, and preventing metabolic disruption (69). Previous studies have shown that shikonin promotes the differentiation of CD4^+^CD25^+^Foxp3^+^ Tregs by inhibiting the AKT/mTOR pathway, which may be a potential mechanism underlying immunosuppressive effects of HMIs on grafts (70). Additionally, previous studies have demonstrated that the pro-inflammatory cytokine TNF-α not only impairs suppressive function of Tregs but also drives their reprogramming into effector T cells (71). Notably, the included studies in this meta-analysis also showed a reduction in TNF-α. Therefore, HMIs may promote the proliferation and functional maintenance of CD4^+^CD25^+^Foxp3^+^ Tregs by suppressing TNF-α expression.

(5) Reducing adverse drug reactions by inhibiting cytochrome P450 (CYP450) activity

The CYP450 system is involved in the metabolism of endogenous and exogenous toxins and is responsible for approximately 90% of the drugs taken by patients (72). CYP450 metabolizes drugs into electrophiles or free radicals, which subsequently cause cellular damage and death, increase adverse drug reactions, or reduce therapeutic efficacy (73). Experimental results from Cui et al. demonstrated that sodium tanshinone A sulfonate significantly reduced the expression of major parts of CYP450 proteins (CYP2E1, CYP1A2, CYP3A4, and CYP2D6). This finding suggests that sodium tanshinone A sulfonate may reduce adverse drug reactions and enhance therapeutic efficacy by inhibiting CYP450 activity, thereby providing protection against hepatic I/R injury (19).

(6) Protecting grafts by inhibiting autophagy induced by ER stress

The endoplasmic reticulum (ER) is crucial for maintaining intracellular calcium and lipid metabolism or homeostasis. Regulated autophagy is considered a compensatory mechanism following ER stress. However, severe or prolonged autophagy may impair cellular function and lead to the irreversible damage (74, 75). Zhang et al. found that upon liver transplantation, steatotic graft-induced liver injury was aggravated, with a significant upregulation in the expression of the ER stress-mediated autophagy marker, the family member with sequence similarity 134 (FAM134B), along with increased expression of other ER stress markers (p-PERK, CHOP, and Bip) and enhanced colocalization of ER tracker (KDEL) and autophagy marker (LC3B). BBR pretreatment suppressed the expression of ER stress markers and exerted protective effects on steatotic grafts by downregulating levels of microtubule-associated protein 1 Light Chain 3 I/Light Chain 3 II (LC3I/LC3II), sequestosome 1 (p62), autophagic protein Beclin1 (Beclin1), and FAM134B. These findings revealed that BBR exerts hepatoprotective effects following liver transplantation by inhibiting ER stress-induced autophagy (30). Additionally, studies have shown that BBR also alleviates myocardial I/R injury in a rat model of I/R injury through the inhibition of ER stress (76).

### 4.1 Strength

Given the remarkable potential of HMIs in immunosuppressive therapy and its unique origin as a traditional herbal extract, its application carries profound traditional values as well as significant contemporary medical relevance. In China, HMI has become a representative adjunctive or alternative intervention in the field of immunomodulation and immune-mediated diseases due to its long history of usage and notable therapeutic potential. Moreover, it’s generally believed that HMI may also serve as a natural nutrition ingredient, thus exhibiting less side effects. Therefore, inclusion of Chinese studies in our meta-analysis not only provides a comprehensive reflection of the global research landscape, but also offers a more holistic perspective for systematically evaluating the efficacy of HMI. Although previous studies have demonstrated that HMI exerts significant therapeutic effects on various immune-related diseases, particularly chronic inflammatory diseases, there is currently no systematic evaluation of its role in mitigating immune-mediated organ transplantation rejection and improving long-term graft function. This gap in research not only limits the broader application of HMI in transplantation medicine but also constrains the deeper exploration and validation of its underlying mechanisms and translational values.

The novelty of this study lies in its systematic collation and analysis of preclinical evidence regarding the effects of HMI in the field of organ transplantation, thereby addressing existing gaps in the literature and offering valuable references for future clinical translation of HMI. This endeavor not only opens new avenues for the integration of herbal interventions in transplantation medicine, but also provides insightful guidance for the development of more effective and safer immunosuppressive treatment strategies.

### 4.2 Limitation

Our study has several limitations (1): Our search results only included English and Chinese literature, however, studies on HMIs could have been found in Japan, Korea and other countries. Although English articles by Japanese scholars have also been cited in this review, articles in minor languages such as Japanese and Korean may not be included in this systemic review, which may introduce linguistic bias. Unfortunately, we do not understand and thus cannot read the literature in a language other than English and Chinese. Fortunately, studies on HMIs or Chinese medicine have been overwhelmingly carried out in China. (2) Although we attempted to conduct a comprehensive search across several major databases, the number of the studies included in our analysis was limited. Given that most of the studies reviewed here were conducted in China, there may be a potential risk of publication bias. (3) The application of HMI in the field of transplantation animal models is currently limited and our exploration has only touched upon a small subset of inflammatory factors involved in the immunopathology and graft rejection, leaving substantial room for further investigation into more specific immune cells and signaling pathways. (4) Some of the included studies did not clearly report the methods of allocation concealment, while in other studies, animals were not randomly selected for outcome measurement, which may limit the reliability of assessing the risk of bias, thus affecting statistical power.

Furthermore, since some outcomes in the current analysis exhibited high heterogeneity, which may compromise the reliability and robustness of the final conclusions, we have identified the following potential sources of heterogeneity:

(1) The inclusion of three organ models of transplantation introduced organ-specific diversity. Each organ exhibits distinct immune rejection patterns. For instance, liver transplantation demonstrated inherent immune tolerance. Merging these results may contribute to heterogeneity. Even for the same type of transplanted organs, distinctions, such as orthotopic vs. heterotopic transplantation or left/right kidney selection, could introduce hemodynamic and physiological variations. (2) In terms of experimental design, different dosing and treatment time may lead to pharmacokinetic differences. Differences in surgical techniques may also affect the degree of graft injury and subsequent immune responses, potentially amplifying the variability of outcomes. (3) Finally, the pooled analysis incorporated both rats and mice, which may differ in immune reactivity and drug metabolism. Taken together, these factors may explain the observed high heterogeneity and highlight the need for future studies to adopt standardized HMI protocols and implement uniform surgical procedures to minimize variability.

### 4.3 Conclusion

This systematic review or meta-analysis highlights the significant therapeutic potential of HMIs in improving organ transplantation outcomes. Our results suggest that HMIs can alleviate acute rejection by improving liver and kidney function, ameliorating pathological damage, inhibiting apoptosis, reducing oxidative damage, and suppressing DC maturation and T cell activation, thereby prolonging graft survival. The identified mechanisms underlying the effects of HMIs may include improving ischemia-reperfusion injury, modulating oxidative stress, suppressing apoptosis, and promoting Treg-mediated immunoregulation. These results provide robust evidence that HMIs may serve as an adjunctive or alternative strategy for improving graft survival and transplantation outcomes while reducing the side effects of conventional immunosuppressants. However, the findings also reveal heterogeneity across studies, which highlights the need for standardized experimental designs, including consistent animal models, HMI dosages, and outcome measures. Furthermore, the mechanisms underlying HMI’s effects are still incompletely understood, and in particular, the specific signaling pathways and key immune cells involved in immunoregulation post-transplantation remain to be defined. Addressing these gaps will require further well-designed and large-scale studies that not only explore the detailed molecular mechanisms, but also assess the translational potential of HMIs in clinical settings. By bridging the gap between traditional medicine and modern immunology, this study underscores the promising role of HMIs in transplantation medicine, thus opening avenues for developing safer and more effective immunosuppressive and anti-inflammatory therapies.

## Data Availability

The original contributions presented in the study are included in the article/supplementary material. Further inquiries can be directed to the corresponding authors.
