# Supplementary material for: Anti-inflammatory and anti-rejection effects of herbal medicine ingredients in organ transplantation: a systematic review and meta-analysis
Source: Front Immunol. 2025 Jun 5;16:1568988. doi: 10.3389/fimmu.2025.1568988 (PMC12176545; doi:10.3389/fimmu.2025.1568988)
Supplement: Supplementary file 1 [file DataSheet1.docx]

**Supplementary Materials**

**Supplementary appendix 1. Search strategy**

**Table 1:** PubMed Search

| **NO** | **Search Details** | **Results** |
| --- | --- | --- |
| #1 | " organ transplantation "[MeSH Terms] | 242,561 |
| #2 | "transplantability"[All Fields] OR "transplantable"[All Fields] OR "transplantated"[All Fields] OR "transplantating"[All Fields] OR "transplantation"[MeSH Terms] OR "transplantation"[All Fields] OR "transplantations"[All Fields] OR "transplanted"[All Fields] OR "transplanting"[All Fields] OR "transplantation"[MeSH Subheading] OR "transplantation s"[All Fields] OR "transplanter"[All Fields] OR "transplanters"[All Fields] OR "transplantion"[All Fields] OR "transplants"[MeSH Terms] OR "transplants"[All Fields] OR "transplant"[All Fields] OR ("transplants"[MeSH Terms] OR "transplantation"[MeSH Terms]) OR "transplants"[MeSH Terms] OR ("graft s"[All Fields] OR "grafted"[All Fields] OR "graftings"[All Fields] OR "transplantation"[MeSH Subheading] OR "transplantation"[All Fields] OR "grafting"[All Fields] OR "transplantation"[MeSH Terms] OR "grafts"[All Fields] OR "transplants"[MeSH Terms] OR "transplants"[All Fields] OR "graft"[All Fields]) OR ("transplants"[MeSH Terms] OR "transplants"[All Fields] OR ("tissue"[All Fields] AND "transplants"[All Fields]) OR "tissue transplants"[All Fields]) OR ("transplants"[MeSH Terms] OR "transplants"[All Fields] OR ("transplant"[All Fields] AND "tissue"[All Fields]) OR "transplant tissue"[All Fields]) OR ("transplants"[MeSH Terms] OR "transplants"[All Fields] OR ("tissue"[All Fields] AND "grafts"[All Fields]) OR "tissue grafts"[All Fields]) OR ("organ transplantation"[MeSH Terms] OR ("organ"[All Fields] AND "transplantation"[All Fields]) OR "organ transplantation"[All Fields]) OR (("graft s"[All Fields] OR "grafted"[All Fields] OR "graftings"[All Fields] OR "transplantation"[MeSH Subheading] OR "transplantation"[All Fields] OR "grafting"[All Fields] OR "transplantation"[MeSH Terms] OR "grafts"[All Fields] OR "transplants"[MeSH Terms] OR "transplants"[All Fields] OR "graft"[All Fields]) AND ("organ"[All Fields] OR "organ s"[All Fields] OR "organism"[All Fields] OR "organism s"[All Fields] OR "organisms"[All Fields] OR "organs"[All Fields])) OR ("organ transplantation"[MeSH Terms] OR ("organ"[All Fields] AND "transplantation"[All Fields]) OR "organ transplantation"[All Fields] OR ("organ"[All Fields] AND "graftings"[All Fields]) OR "organ graftings"[All Fields]) OR (("transplantability"[All Fields] OR "transplantable"[All Fields] OR "transplantated"[All Fields] OR "transplantating"[All Fields] OR "transplantation"[MeSH Terms] OR "transplantation"[All Fields] OR "transplantations"[All Fields] OR "transplanted"[All Fields] OR "transplanting"[All Fields] OR "transplantation"[MeSH Subheading] OR "transplantation s"[All Fields] OR "transplanter"[All Fields] OR "transplanters"[All Fields] OR "transplantion"[All Fields] OR "transplants"[MeSH Terms] OR "transplants"[All Fields] OR "transplant"[All Fields]) AND ("organ"[All Fields] OR "organ s"[All Fields] OR "organism"[All Fields] OR "organism s"[All Fields] OR "organisms"[All Fields] OR "organs"[All Fields])) OR "transplants"[MeSH Terms] OR "transplants"[MeSH Terms] OR "transplants"[MeSH Terms] OR "transplants"[MeSH Terms] OR "organ transplantation"[MeSH Terms] OR "organ transplantation"[MeSH Terms] | 1,236,480 |
| #3 | (("Herbal Medicine"[Mesh]) OR "Medicine, Chinese Traditional"[Mesh]) OR "Drugs, Chinese Herbal"[Mesh] | 73,623 |
| #4 | "herbal medicine"[MeSH Terms] OR ("herbal"[All Fields] AND "medicine"[All Fields]) OR "herbal medicine"[All Fields] OR "herbalism"[All Fields] OR "herbal"[All Fields] OR "herbals"[All Fields] OR "herbal medicine"[MeSH Terms] OR "medicine, chinese traditional"[MeSH Terms] OR ("medicine, chinese traditional"[MeSH Terms] OR ("medicine"[All Fields] AND "chinese"[All Fields] AND "traditional"[All Fields]) OR "chinese traditional medicine"[All Fields] OR ("traditional"[All Fields] AND "medicine"[All Fields] AND "chinese"[All Fields]) OR "traditional medicine chinese"[All Fields]) OR ("medicine, chinese traditional"[MeSH Terms] OR ("medicine"[All Fields] AND "chinese"[All Fields] AND "traditional"[All Fields]) OR "chinese traditional medicine"[All Fields] OR ("chinese"[All Fields] AND "traditional"[All Fields] AND "medicine"[All Fields])) OR "medicine, chinese traditional"[MeSH Terms] OR "medicine, chinese traditional"[MeSH Terms] OR ("medicine, chinese traditional"[MeSH Terms] OR ("medicine"[All Fields] AND "chinese"[All Fields] AND "traditional"[All Fields]) OR "chinese traditional medicine"[All Fields] OR ("traditional"[All Fields] AND "chinese"[All Fields] AND "medicine"[All Fields]) OR "traditional chinese medicine"[All Fields]) OR ("medicine, chinese traditional"[MeSH Terms] OR ("medicine"[All Fields] AND "chinese"[All Fields] AND "traditional"[All Fields]) OR "chinese traditional medicine"[All Fields] OR ("chinese"[All Fields] AND "medicine"[All Fields] AND "traditional"[All Fields]) OR "chinese medicine traditional"[All Fields]) OR "medicine, chinese traditional"[MeSH Terms] OR "drugs, chinese herbal"[MeSH Terms] OR ("drugs, chinese herbal"[MeSH Terms] OR ("drugs"[All Fields] AND "chinese"[All Fields] AND "herbal"[All Fields]) OR "chinese herbal drugs"[All Fields] OR ("chinese"[All Fields] AND "drugs"[All Fields] AND "plant"[All Fields])) OR ("drugs, chinese herbal"[MeSH Terms] OR ("drugs"[All Fields] AND "chinese"[All Fields] AND "herbal"[All Fields]) OR "chinese herbal drugs"[All Fields] OR ("chinese"[All Fields] AND "herbal"[All Fields] AND "drugs"[All Fields])) OR "drugs, chinese herbal"[MeSH Terms] OR "drugs, chinese herbal"[MeSH Terms] OR ("drugs, chinese herbal"[MeSH Terms] OR ("drugs"[All Fields] AND "chinese"[All Fields] AND "herbal"[All Fields]) OR "chinese herbal drugs"[All Fields] OR ("herbal"[All Fields] AND "drugs"[All Fields] AND "chinese"[All Fields])) OR ("drugs, chinese herbal"[MeSH Terms] OR ("drugs"[All Fields] AND "chinese"[All Fields] AND "herbal"[All Fields]) OR "chinese herbal drugs"[All Fields] OR ("plant"[All Fields] AND "extracts"[All Fields] AND "chinese"[All Fields])) OR "drugs, chinese herbal"[MeSH Terms] OR "drugs, chinese herbal"[MeSH Terms] OR ("drugs, chinese herbal"[MeSH Terms] OR ("drugs"[All Fields] AND "chinese"[All Fields] AND "herbal"[All Fields]) OR "chinese herbal drugs"[All Fields] OR ("chinese"[All Fields] AND "plant"[All Fields] AND "extracts"[All Fields]) OR "chinese plant extracts"[All Fields]) OR ("drugs, chinese herbal"[MeSH Terms] OR ("drugs"[All Fields] AND "chinese"[All Fields] AND "herbal"[All Fields]) OR "chinese herbal drugs"[All Fields] OR ("extracts"[All Fields] AND "chinese"[All Fields] AND "plant"[All Fields])) OR "drugs, chinese herbal"[MeSH Terms] | [238,062](https://pubmed.ncbi.nlm.nih.gov/?term=%28%28%28%28%28%28%28%28%28%28%28%28%28%28%28%28%28%28%28%28%28Herbalism%29+OR+%28Herbalism%5BMeSH+Terms%5D%29%29+OR+%28Traditional+Medicine%2C+Chinese%5BMeSH+Terms%5D%29%29+OR+%28Traditional+Medicine%2C+Chinese%29%29+OR+%28Chinese+Traditional+Medicine%29%29+OR+%28Chinese+Traditional+Medicine%5BMeSH+Terms%5D%29%29+OR+%28Traditional+Chinese+Medicine%5BMeSH+Terms%5D%29%29+OR+%28Traditional+Chinese+Medicine%29%29+OR+%28Chinese+Medicine%2C+Traditional%29%29+OR+%28Chinese+Medicine%2C+Traditional%5BMeSH+Terms%5D%29%29+OR+%28Chinese+Drugs%2C+Plant%5BMeSH+Terms%5D%29%29+OR+%28Chinese+Drugs%2C+Plant%29%29+OR+%28Chinese+Herbal+Drugs%29%29+OR+%28Chinese+Herbal+Drugs%5BMeSH+Terms%5D%29%29+OR+%28Herbal+Drugs%2C+Chinese%5BMeSH+Terms%5D%29%29+OR+%28Herbal+Drugs%2C+Chinese%29%29+OR+%28Plant+Extracts%2C+Chinese%29%29+OR+%28Plant+Extracts%2C+Chinese%5BMeSH+Terms%5D%29%29+OR+%28Chinese+Plant+Extracts%5BMeSH+Terms%5D%29%29+OR+%28Chinese+Plant+Extracts%29%29+OR+%28Extracts%2C+Chinese+Plant%29%29+OR+%28Extracts%2C+Chinese+Plant%5BMeSH+Terms%5D%29&sort=) |
| #5 | #1 or #2 | 1,236,480 |
| #6 | #3 or #4 | 238,151 |
| #7 | ("rodent s"[All Fields] OR "rodentia"[MeSH Terms] OR "rodentia"[All Fields] OR "rodent"[All Fields] OR "rodents"[All Fields] OR "rodentia"[MeSH Terms] OR "mice"[MeSH Terms] OR "mice"[All Fields] OR "mice"[MeSH Terms] OR "rats"[MeSH Terms] OR "rats"[MeSH Terms]) AND (animal[Filter]) | 3,645,579 |
| #8 | #5 and #6 and #7 | 2,063 |

**Table 2:** EMbase Search

| **NO** | **Search Details** | **Results** |
| --- | --- | --- |
| #1 | 'organ transplantation'/exp OR 'organ transplantation':ab,ti OR 'organ grafting':ab,ti OR 'organ transplantations':ab,ti OR 'organ grafting':ab,ti OR 'grafting, Organ ':ab,ti OR 'graft ':ab,ti OR 'grafting ':ab,ti OR 'transplant ':ab,ti OR ' Tissue Transplants ':ab,ti | 988,645 |
| #2 | 'chinese medicine'/exp OR 'chinese medicine' OR 'chinese herbal medicine':ab,ti OR 'chinese traditional medicine':ab,ti OR 'medicine, chinese traditional':ab,ti OR 'traditional chinese medicine':ab,ti OR 'chinese medicine':ab,ti OR ' herbal Ingredients ':ab,ti | 269,945 |
| #3 | 'rodent'/exp OR 'rodent' OR 'mouse'/exp OR 'mouse' OR 'rat'/exp OR 'rat' | 4,676,313 |
| #4 | #1 AND #2 AND #3 | 548 |

Table 3: Web of Science Search

| **NO** | **Search Details** | **Results** |
| --- | --- | --- |
| #1 | TS=(organ transplantation OR Organ Grafting OR Organ Transplantations OR Transplantations, Organ OR Graftings, Organ OR transplant OR Grafts OR Tissue Transplants OR Graft) | 1,121,642 |
| #2 | TS=(Chinese herbal medicine OR Chinese traditional medicine OR medicine, Chinese traditional OR traditional Chinese medicine OR Chinese medicine OR Chinese medicine OR herbal Ingredients) | 212,544 |
| #3 | TS=(rodent OR rodentia OR mouse OR mice OR rat OR animal) | 21,778,285 |
| #4 | #1 AND #2 AND #3 | 1989 |

**Table 4:** Cochrane Library Search

| **NO** | **Search Details** | **Results** |
| --- | --- | --- |
| #1 | MeSH descriptor: [Organ Transplantation] explode all trees | 7620 |
| #2 | MeSH descriptor: [Medicine, Chinese Traditional] explode all trees | 1,778 |
| #3 | (Transplant):ti,ab,kw OR (Grafts):ti,ab,kw OR (Graft):ti,ab,kw OR (Tissue Transplants):ti,ab,kw OR (Tissue Grafts):ti,ab,kw OR (Transplantation, Organ):ti,ab,kw OR (Organ Transplantations):ti,ab,kw OR (Organ Graftings):ti,ab,kw OR (Graftings, Organ):ti,ab,kw OR (Transplantations):ti,ab,kw | 68592 |
| #4 | (Chinese Medicine):ti,ab,kw OR ("Chinese herbal medicine"):ti,ab,kw OR ("traditional Chinese medicine theory"):ti,ab,kw OR ("Chinese herbal medicine"):ti,ab,kw OR (Chinese folk-medicine):ti,ab,kw | 16763 |
| #5 | MeSH descriptor: [Mice] explode all trees | 2,050 |
| #6 | (mouse):ti,ab,kw OR (mice):ti,ab,kw OR (rodent):ti,ab,kw OR (rats):ti,ab,kw OR (animal):ti,ab,kw | 31,005 |
| #7 | #1 or #3 | 68592 |
| #8 | #2 or #4 | 16992 |
| #9 | #5 or #6 | 31,005 |
| #10 | #7 and #8 and #9 | 5 |

**Table 5:** CNKI Search

| **NO** | **Search Details** | **Results** |
| --- | --- | --- |
| #1 | (SU%= '中药单体' OR SU%= '中药单体化合物' OR SU%= '中药单体及其制剂' OR SU%= '中药单体及复方' OR SU%= ‘黄芩苷’OR SU%= ‘人参皂苷’ OR SU%= ‘丹参酮’ OR SU%= ‘ 甘草酸 OR’ SU%= ‘ 苏木素’ OR SU%= ‘ 紫草素’ OR SU%= ‘ 青蒿素’ OR SU%= ‘ 黄连素’ OR SU%= ‘莪术酮’ OR SU%= ‘ 阿魏酸’ OR SU%= ‘灵芝多糖’ OR SU%= ‘ 茯苓多糖’OR SU%= ‘山奈酚’) AND (SU%= '器官移植' OR SU%= '移植' OR SU%= '异体器官移植') AND (SU%='鼠' OR SU%= '动物') | 153 |

**Table 6:** Wanfang Search

| **NO** | **Search Details** | **Results** |
| --- | --- | --- |
| #1 | 主题:(器官移植 or 同种异体器官 or移植) | 14,547 |
| #2 | 主题:( 中药单体or黄芩苷or人参皂苷 or丹参酮 or 甘草酸 or 苏木素 or 紫草素 or 青蒿素 or 黄连素 or莪术酮 or 阿魏酸 or灵芝多糖 or 茯苓多糖 or山奈酚) | 33115 |
| #3 | 主题:(鼠 or 动物) | 1,810,646 |
| #4 | #1 and #2 and #3 | 0 |

**Table 7:** CBM Search

| NO | Search Details | Results |
| --- | --- | --- |
| #1 | 器官移植 or 同种异体器官 or移植 | 55545 |
| #2 | 中药单体or 单体or黄芩苷or人参皂苷 or丹参酮 or 甘草酸 or 苏木素 or 紫草素 or 青蒿素 or 黄连素 or莪术酮 or 阿魏酸 or灵芝多糖 or 茯苓多糖or山奈酚 | [32816](javascript:void(0);) |
| #3 | 鼠 or 动物 | 8566355 |
| #4 | #1 and #2 and #3 | 176 |

**Table 8:** VIP Search

| NO | Search Details | Results |
| --- | --- | --- |
| #1 | M=(器官移植 or 同种异体器官 or移植) | 12,777 |
| #2 | M=(中药单体or单体or黄芩苷or人参皂苷 or丹参酮 or 甘草酸 or 苏木素 or 紫草素 or 青蒿素 or 黄连素 or莪术酮 or 阿魏酸 or灵芝多糖 or 茯苓多糖or山奈酚) | 12,697 |
| #3 | M=(鼠 or 动物) | 660,180 |
| #4 | #1 and #2 and #3 | 0 |
