# Supplementary material for: Anti-inflammatory and anti-rejection effects of herbal medicine ingredients in organ transplantation: a systematic review and meta-analysis
Source: Front Immunol. 2025 Jun 5;16:1568988. doi: 10.3389/fimmu.2025.1568988 (PMC12176545; doi:10.3389/fimmu.2025.1568988)
Supplement: Supplementary file 2 [file Table1.docx]

| **Supplementary Table 1. Characteristics of the study** | | | | | | | | | |
| --- | --- | --- | --- | --- | --- | --- | --- | --- | --- |
| Study | Donor Animal models | Recipient Animal models | HMI group/  Control group | Method of establishing model | Intervention  type | Duration | Intervention  (HMI group) | Intervention  (control group) | Outcomes |
| Zhou,J.B, 2007 | SD rat | SD rat | 6/6 | OLT | 2ml of  40mg/kg  EGb and  Saline mixture | 1hour before OLT | Dorsal penile vein injection | 2 ml physiological saline | ④⑤⑥⑦ |
| Bai,J.H,2008 | SD rat | Wistar rat | 5/5 | OLT | Artemisinin olive oil 150mg/(kg·d) | 10 days | Oral gavage | 2ml olive oil | ②⑥⑦⑧⑲⑳㉑㉓ |
| Jing, H, 2008 | Lewis rat | Brown Norway rat | 12/12 | OLT | Emodin 50 mg/(kg·d) | 7 days | Intraperitoneal injection | 0.5 ml physiological saline | ①⑬ |
| Meng,K.W,2009 | SD rat | Wistar rat | 18/17 | OLT | Emodin 250 mg/(kg·d) | 7 days | Intraperitoneal injection | Equal volume physiological saline | 1. ②③⑬ |
| Ding,H.X, 2010 | SD rat | Wistar rat | 5/5 | OLT | Artemisinin 250 mg/(kg·d) | 9 days | Oral gavage | 2ml olive oil | ②⑥⑦⑧ |
| Cui,M.H,2016 | SD rat | SD rat | 12/6 | 1/2 OLT | Sodium tanshinone A sulfonate 5 mg/(kg·d) | 14 days | Tail vein injection | Equal volume physiological saline | ⑥⑦ |
| Mu,H.N,2018 | SD rat | SD rat | 29/29 | OLT | CA 15 mg/kg/h | 30 min before OLT till 120 min after reperfusion | Intraperitoneal injection | Equal volume physiological saline | ④⑤⑥⑦㉒ |
| Zhang,N,2019 | Wistar rat | Wistar rat | 6/6 | LT | BBR 200 mg/(kg·d) | 1 week before LT | Oral gavage | Same conditions without any treatment | ④⑤⑪⑫ |
| He,B,2016 | SD rat | SD rat | 20/20 | bilaterally nephrectomized | 8 mg/100 g body weight of a solution of saponins | 48, 24 and 0.5 h before being anesthetized | Intraperitoneal injection | Equal volume physiological saline | ③⑨⑩⑪⑫⑬㉒ |
| Gao,C,2007 | Fisher 344 rat | Lewis rat | 15/15 | unilaterally right nephrectomized | Ligustrazine (100mg/kg /d) combined with CsA(10mg/kg /d) | / | / | CsA(10mg/kg /d) | ②③⑨⑩⑪ |
| Chen,S.X,2017 | SD rat | SD rat | 9/5 | unilaterally left nephrectomized | 10%  tanshinone ⅡA  5 mL/kg | 7 days | Oral gavage | Equal volume physiological saline | ⑨⑩⑪⑫ |
| Zhao,F.2020 | Wistar rat | SD rat | 10/10 | Ono's technique heterotopic cardiac transplantation | ethyl acetate extract solution of hematoxylin 10 mL/kg | 7 days | Oral gavage | Equal volume physiological saline | ⑬㉙ |
| Zhang,G.W,2004 | Wistar rat | SD rat | 10/10 | Ono's technique  cardiac allograft implantation | EEPCW 5 ml/kg/d | 1 day before transplantation to POD 7 or the day of heart arrest | Inject into the stomach | Olive oil 8 ml/kg/d | 1. ㉔㉚ |
| Zhang,L,2009 | Wistar rat | SD rat | 8/8 | Ono's technique  cardiac allograft implantation | 50 mg/kg asarinin | 1 day before transplantation to POD 7 or the day of heart arrest | Oral gavage | 8 ml/kg olive oil | ①⑲⑳㉑㉓ |
| Ma,Y.H,2021 | BALB/c mouse | C57BL/6 mouse | 3/3 | Heterotopic cardiac transplantation | 5 mg/kg of berberine | 10 days | Intraperitoneal injection | Equal volume physiological saline | ⑤⑮⑯⑰⑱ |
| Zheng,S.H,2021 | BALB/c mouse | C57BL/6 mouse | 5/5 | Heart transplant cuff model | 50 mg/kg/d of matrine | POD 0 to the allograft was rejected | Intraperitoneal injection | Equal volume physiological saline | ⑭⑮⑯⑰⑱㉕㉖㉗㉘ |
| Liu,Y,2008 | C57BL/6 mouse | BALB/c mouse | 6/6 | Heterotopic cardiac transplantation | 3 mg/kg/d of TPT | POD 0–7, 9, 11, 13 and 15 | Administered orally | No treatment | 1. ⑭⑲⑳㉑㉛ |
| Yamamoto,2022 | C57BL/6 mouse | CBA mouse | 5/5 | Heterotopic cardiac transplantation | 2.0 mg/d of GA | 7 days | Intraperitoneal injection | Equal volume physiological saline | ⑭ |
| 1. : MST; ②:Banff schema; ③:Bcl-2; ④: IL-1β; ⑤: TNF-α; ⑥: ALT; ⑦: AST; ⑧:TBIL; ⑨: BUN; ⑩:Scr; ⑪:SOD; ⑫:MDA; ⑬:AI;⑭: CD4^+^ Foxp3^+^ Treg% in SPCs; ⑮: CD4^+^% in SPCs   ⑯:CD8^+^% in SPCs; ⑰:CD4^+^% in LNCs; ⑱:CD8^+^% in LNCs; ⑲: IFN-γ; ⑳: IL-4; ㉑: IL-10; ㉒: Bcl-2/Bax; ㉓:IL-2;㉔: CD3^+^%;㉕:CD11c ^+^ CD86 ^+^% in LNCs; ㉖:CD11c ^+^ CD80 ^+^% in LNCs; ㉗:CD11c ^+^ CD86 ^+^% in SPCs;㉘:CD11c ^+^ CD80 ^+^% in SPCs; ㉙:caspase-3; ㉚: CD4^+/^ CD8^+^; ㉛：IL-12  OLT: Orthotopic liver transplantation; LT: liver transplantation; POD: Post-operative day; SD: Spraque Dawley | | | | | | | | | |
